# Differential sensitivity to speech rhythms in young and older adults

**DOI:** 10.3389/fpsyg.2023.1160236

**Published:** 2023-05-12

**Authors:** Dylan V. Pearson, Yi Shen, J. Devin McAuley, Gary R. Kidd

**Affiliations:** ^1^Department of Speech, Language, and Hearing Sciences, Indiana University, Bloomington, IN, United States; ^2^Department of Speech and Hearing Sciences, University of Washington, Seattle, WA, United States; ^3^Department of Psychology, Michigan State University, East Lansing, MI, United States

**Keywords:** hearing, speech, rhythm, aging, attention

## Abstract

Sensitivity to the temporal properties of auditory patterns tends to be poorer in older listeners, and this has been hypothesized to be one factor contributing to their poorer speech understanding. This study examined sensitivity to speech rhythms in young and older normal-hearing subjects, using a task designed to measure the effect of speech rhythmic context on the detection of changes in the timing of word onsets in spoken sentences. A temporal-shift detection paradigm was used in which listeners were presented with an intact sentence followed by two versions of the sentence in which a portion of speech was replaced with a silent gap: one with correct gap timing (the same duration as the missing speech) and one with altered gap timing (shorter or longer than the duration of the missing speech), resulting in an early or late resumption of the sentence after the gap. The sentences were presented with either an intact rhythm or an altered rhythm preceding the silent gap. Listeners judged which sentence had the altered gap timing, and thresholds for the detection of deviations from the correct timing were calculated separately for shortened and lengthened gaps. Both young and older listeners demonstrated lower thresholds in the intact rhythm condition than in the altered rhythm conditions. However, shortened gaps led to lower thresholds than lengthened gaps for the young listeners, while older listeners were not sensitive to the direction of the change in timing. These results show that both young and older listeners rely on speech rhythms to generate temporal expectancies for upcoming speech events. However, the absence of lower thresholds for shortened gaps among the older listeners indicates a change in speech-timing expectancies with age. A further examination of individual differences within the older group revealed that those with better rhythm-discrimination abilities (from a separate study) tended to show the same heightened sensitivity to early events observed with the young listeners.

## Introduction

Difficulty hearing speech in complex environments is one of the most common problems reported by older listeners. Although hearing loss accounts for much of this difficulty, many studies have found that audibility cannot fully account for differences in speech-in-noise performance (Gordon-Salant and Fitzgibbons, 1993; Humes et al., 1994; Pichora-Fuller et al., 1995; Frisina and Frisina, 1997; Gordon-Salant, 2005; Vermiglio et al., 2012; Carroll et al., 2016; Holmes and Griffiths, 2019). Decreases in cognitive abilities and temporal-processing abilities are two of the most often cited factors accounting for increased difficulty understanding speech in noise among older listeners (Humes et al., 2013; Füllgrabe et al., 2015; Gieseler et al., 2017; Nuesse et al., 2018). Among cognitive abilities, working memory and attentional control have often been found to be important for speech understanding, especially under difficult listening conditions (Humes, 2007; Akeroyd, 2008; Houtgast and Festen, 2008; Humes and Dubno, 2010; Humes et al., 2013; Tierney et al., 2020). Although there is good agreement that temporal processing abilities also have a significant influence on the ability to understand speech in noise, especially in the presence of modulated noise, competing speech, or other time-varying sounds (Sommers and Humes, 1993; Dubno et al., 2002; George et al., 2006, 2007; Houtgast and Festen, 2008; Humes and Dubno, 2010; Fitzgibbons and Gordon-Salant, 2010a,b), it has been difficult to establish a clear connection between specific measures of temporal processing ability and speech understanding. Among young normal-hearing (NH) listeners, differences in neither spectral nor temporal resolving power (measured with nonspeech stimuli) account for individual differences in speech understanding in Gaussian noise (Karlin, 1942; Surprenant and Watson, 2001; Watson and Kidd, 2002; Kidd et al., 2007). Among older listeners, the situation is less clear; age-related deficits in termporal sensitivity that may be linked to speech understanding are frequently found (e.g., Fitzgibbons and Gordon-Salant, 1994, 2004, 2011; Schneider et al., 1994; Snell and Frisina, 2000; Gordon-Salant and Fitzgibbons, 2004; Lister and Tarver, 2004; Humes et al., 2009, 2013; Gallego Hiroyasu and Yotsumoto, 2020; Humes, 2021), but the causal role of temporal-processing deficits is difficult to determine, due to correlations with other age-related factors, such as hearing loss, cognitive decline and a general perceptual slowing (see Schneider et al., 2005; Humes and Dubno, 2010; Humes et al., 2013). Notably, temporal processing deficits among the elderly tend to be greater when measured in the context of a temporal sequence than with isolated events (e.g., Fitzgibbons and Gordon-Salant, 1995; Gordon-Salant and Fitzgibbons, 1999; Fitzgibbons and Gordon-Salant, 2001), suggesting that the primary influence of temporal processing abilities on speech understanding may be related to the ability to attentionally track temporal patterns, rather than differences in basic temporal resolving power.

Although many studies have shown a decrease in temporal processing abilities with age, relatively little is known about the effect of age on sensitivity to suprasegmental timing patterns or speech rhythm. It is clear that prosody is important for speech understanding (see Cutler and Swinney, 1987; Cutler et al., 1997; Fletcher, 2010), and that temporal aspects of prosody influence speech perception in young and older listeners (e.g., Hoyte et al., 2009). Both word segmentation and phoneme identification are affected by the manipulation of suprasegmental timing (Martin, 1972; Kidd, 1989; Cutler and Mehler, 1993; Dilley and McAuley, 2008). However, few studies have measured sensitivity to changes in the timing of speech events in running speech in both young and older adults. This is the focus of the present study.

The temporal envelope of naturally produced speech exhibits a quasi-rhythmic structure which provides sufficient predictability for listeners to create temporal expectations that influence speech perception (Kidd, 1989; Dilley and McAuley, 2008). Similarly, a more regular rhythmic structure in an auditory signal has been demonstrated to facilitate perception (Aubanel et al., 2016; Wang et al., 2018; Shen and Pearson, 2019), and degrading the natural rhythm of speech has been found to decrease speech understanding in noise and multi-talker backgrounds (McAuley et al., 2020, 2021). However, it is not clear how the ability to use the rhythmic regularity of speech to aid speech understanding may change with age, or how important this ability is for speech understanding compared to other age-related changes in temporal processing or cognitive abilities.

A potential explanation of how sensitivity to timing and rhythmic structure affects speech understanding is based on dynamic attending theory (DAT, see Jones, 1976; Jones and Boltz, 1989; Large and Jones, 1999). DAT proposes that rhythms in the environment (i.e., stimulus rhythms) serve to entrain (synchronize) natural temporal fluctuations in listeners’ attention. This stimulus-driven attentional synchronization focuses pulses of attentional energy at periodic time intervals that align with rhythmically salient points in the stimulus, thus facilitating the perception of events that occur at these time points. Support for DAT has been found in studies showing better discrimination and detection of events that occur at rhythmically expected times than those that are early or late relative to rhythmic expectations (McAuley and Kidd, 1998; Jones et al., 2002; McAuley and Jones, 2003; Jones and McAuley, 2005; Miller et al., 2013; McAuley and Fromboluti, 2014).

Speech rhythm entrainment can also help explain some effects of speech context where expectations set by the temporal context of preceding speech can influence word segmentation and lexical processing (Kidd, 1989; Dilley and McAuley, 2008; Morrill et al., 2014; Baese-Berk et al., 2019). In these studies, changes in speech rhythms or tempos established early in a spoken sentence create expectancies that influence the perception of later-occurring words or syllables, despite the absence of temporal changes in their local context. Several neurophysiological studies have also provided support for DAT by showing that synchrony between cortical oscillations and speech rhythms is important for the understanding of speech, and for the separation of a single speech stream from background sounds (e.g., Ahissar et al., 2001; Luo and Poeppel, 2007; Giraud and Poeppel, 2012; Golumbic et al., 2012; see Peelle and Davis, 2012, for a review).

The current study provides another test of DAT using a task that measures listeners’ ability to detect deviations from the natural timing of events in spoken sentences with intact or rhythmically altered timing prior to the temporal deviation. The task is to judge whether a spoken sentence that is briefly inaudible (replaced by silence) continues at the correct time when audibility returns. The onset of the sentence continuation after the silent period occurs either at the correct time (as though the sentence had continued without interruption), or it is temporally shifted, occurring slightly earlier or later than in the intact sentence. The task is performed with sentences that are either rhythmically intact or rhythmically altered prior to the silent interruption. A comparison of judgment accuracy with a rhythmically altered vs. intact early sentence provides a measure of the listener’s ability to use the speech rhythm in the earlier part of the sentence to predict the timing of later-occurring speech events. The study includes both young and older normal-hearing listeners to determine whether the ability to use speech rhythm to predict the timing of upcoming speech events changes with age.

According to DAT, the natural rhythms of speech facilitate attentional entrainment and lead to temporal expectations about the onsets of upcoming speech events. Thus, if the predictable speech rhythms are disrupted early in a spoken sentence, listeners will have difficulty anticipating the onsets of later-occurring events in the sentence. Therefore, the ability to detect a temporal shift in sentence timing after the silent interruption should be degraded when the rhythm of the preceding speech is altered. A comparison of young and older listeners’ abilities to detect temporal deviations with intact and rhythmically altered sentence contexts will help to determine the extent to which older listeners’ speech understanding problems may be due to changes in the ability to use speech rhythms to predict the onset of upcoming speech events. If older listeners are less able to use rhythmic context to guide temporal expectations, their performance should be poorer with rhythmically intact sentence contexts and they should be less affected by rhythmic alterations. Additionally, an examination of sensitivity to late onsets vs. early onsets after the brief silent period will help us evaluate the symmetry of the temporal expectancies and may reveal differences in the temporal expectancies generated by young vs. older listeners. Earlier studies with nonspeech stimuli have found an asymmetry in the perception of unexpectedly early and late events (e.g., Halpern and Darwin, 1982; McAuley and Kidd, 1998; McAuley and Fromboluti, 2014; Di Luca and Rhodes, 2016). In the present context, a comparison of detection accuracy for early and late deviations has the potential to provide insight into the nature of rhythm-based temporal expectancies in speech perception, and to show how rhythmic sensitivity may change with age.

Finally, most of the older subjects in this study participated in a large test battery as part of a separate study. The test battery was designed to examine the relation between rhythm perception and speech perception using a variety of speech and non-speech measures. Three tasks were selected from the battery based on their focus on temporal and rhythm processing and possible connection with the perception of speech rhythm. The first was a gap detection task (GAP), where listeners were given a fixed set of gap detection trials and performance was recorded as percent correct. The second was a rhythm discrimination (RD) task, where listeners were presented two rhythms and made a same/different judgment, and discrimination sensitivity was measured using *d*’. The third was a synchronization and continuation (S&C) tapping task where listeners tapped in synchrony with an isochronous auditory stimulus presented at different tempi and then continued tapping at the same tempo after the stimulus stopped. These tasks were used to determine whether these temporal/rhythm abilities might be associated with the older listeners’ sensitivity to sentence timing as measured in the present study. A measure of working memory from the earlier test battery was also included to evaluate a non-rhythmic cognitive ability as a predictor of performance in the current study.

## Materials and methods

### Participants

Twenty-one native English speakers were recruited from the Bloomington, Indiana area to participate in the experiment. The young cohort consisted of 11 participants (7 female) ranging from 18 to 26 years (mean age: 20.8) recruited from the student population at Indiana University. The older cohort consisted of 10 participants (7 female) ranging from 59 to 71 years (mean age: 63.2). All listeners had normal hearing as defined by audiometric thresholds equal to or better than 25 dB HL (ANSI, 2004) from 250 through 8,000 Hz in both ears, with the exception of one older listener who had a hearing loss at 8,000 Hz of 35 (right ear) and 55 (left ear) dB HL. [The definition of normal hearing used here is consistent with that specified by WHO (1991)]. Older subjects showed no signs of cognitive impairment (Mini Mental Status Exam, MMSE, > 25; Folstein et al., 1975), and all subjects had English as their native language.

Listeners were compensated at an hourly rate for their participation and informed consent was obtained prior to data collection. Ethical approval (IRB#2007541750) was obtained from the institutional review board at Indiana University.

### Stimuli

**Figure 1:**
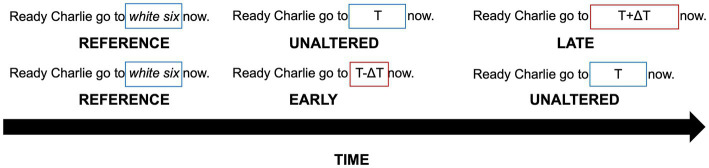
A diagram showing a late-onset trial **(top)** and an early-onset trial **(bottom)**. An intact reference sentence is presented first, followed by two comparison sentences; one with a temporally unaltered gap and one with a temporally altered gap, presented in random order. *T* represents the duration of the “*white six*” portion of the sentence (blue boxes) and *Δ**T* represents the change in duration (red boxes indicate durations that have been altered by **Δ*T*).

This study used the sentence “Ready Charlie go to white six now” from the Coordinate Response Measure (CRM) corpus (Bolia et al., 2000), presented at 68 dB SPL. The sentence was read by two different speakers (1 male, 1 female) which alternated across trials. Each trial consisted of a reference sentence (the intact original sentence), followed by two comparison sentences in which the “*white six*” portion of the sentence was replaced with a silent gap. One comparison sentence had a silent gap equal to the duration of the missing speech and the other comparison sentence included a slightly longer or shorter silent gap. The task was to indicate which comparison pattern had the incorrect silent gap, resulting in the word “now” occurring too late or too early (see Figure 1). The order of the two comparison patterns (correct vs. incorrect silent duration) was randomized across trials. The inter-stimulus interval between sentences randomly varied between 400 and 800 ms to prevent any potential temporal expectation of the “now” onset based on timing regularity across sentences within a trial. The unaltered silent gap, *T*, between “to” and “now” (with “white six” removed) was 1,661 ms for the male speaker and 1,603 ms for the female speaker. The temporally altered comparison sentence had either a shortened gap duration (*T*-Δ*T*) or a lengthened duration (*T* + Δ*T*), leading to the onset of “*now*” occurring unexpectedly early or late, respectively, relative to the timing of the reference (unaltered) sentence. The values of Δ*T* for the early-onset and late-onset conditions were independently varied adaptively (as a proportion of the reference duration) within a block of trials.

To evaluate the influence of the speech rhythm in the early part of the sentences on the ability to detect temporal shifts in the onsets of later-occurring words, this study included a condition in which sentence rhythm was altered using a rhythm alteration that maintained intelligibility (and some degree of naturalness) while disturbing the natural speech rhythm. The rhythm alteration was applied to the early portion of the reference and comparison sentences (i.e., “Ready Charlie go to”) by first dividing that portion of the sentence into 50-ms frames. The speech in these frames was then independently compressed or expanded according to a sinusoidal modulator. The adjusted new frame duration relative to the original frame duration (50 ms) is given by:


$$\frac{\text{New}\;\text{Frame Duration}}{\text{Original Frame Duration}}=1+m\;\sin\left(2\pi f_{m}t+\phi \right),$$

where *m*, *f_m_*, and *ϕ* are the modulation depth, modulation rate and the initial phase of the modulator, respectively.

The modulation depth dictated the degree of rhythm alteration. At a modulation depth of 100%, the new frame duration would be double the original frame duration at the peak of the sinusoidal modulator and it would be compressed to 0 ms at the trough of the modulator. At a modulation depth of 0%, the new frame duration would always be equal to the original frame duration. In the altered-rhythm condition in the current experiment, the modulation depth was set to 75%. This value was selected, based on earlier work, to introduce a salient rhythm alteration while maintaining good intelligibility (McAuley et al., 2020).

**Figure 2:**
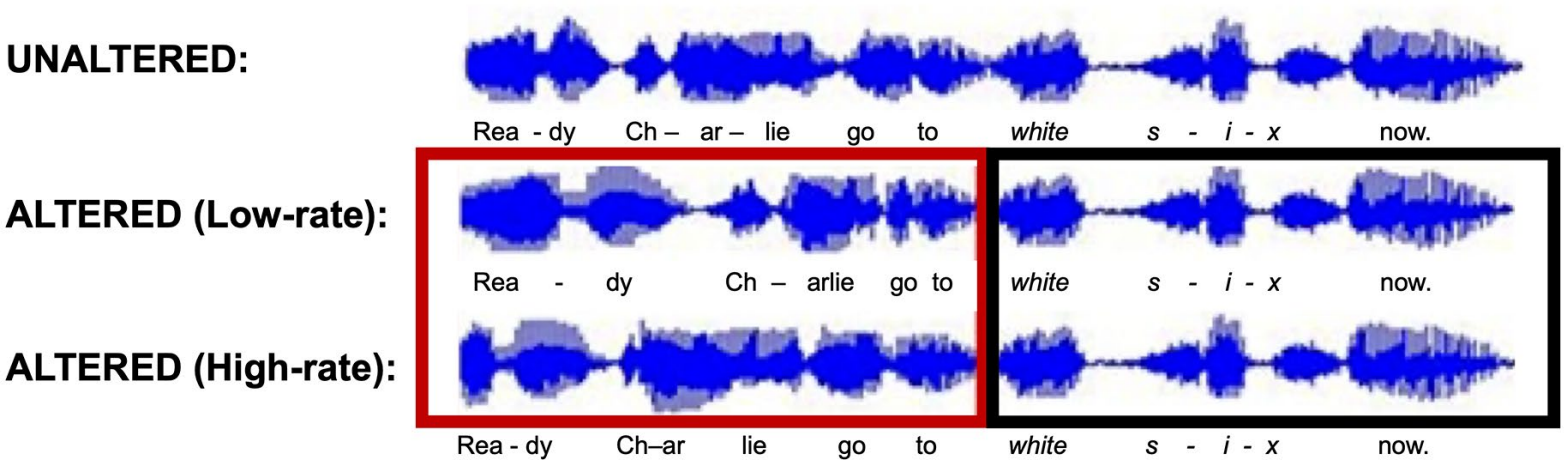
Example waveforms of the reference sentence in each of the three rhythm conditions. The red box highlights the portion of the sentences that are rhythmically altered. The black box indicates the unaltered portion.

The modulation rate determined the frequency of the alternating compression and expansion within the early portion of the sentence. For this experiment, two modulation rates were used: a low-rate, which consisted of one modulator cycle, and a high-rate, which consisted of three modulator cycles. Thus, there was either one cycle of sinusoidal shortening and lengthening, or three cycles of shortening and lengthening in the early portion of the sentence. Figure 2 shows the effect of the high- and low-rate rhythm alteration on the waveform of the sentence. In this example, the Altered (Low-rate) sentence has the lengthening period at the start of the sentence, which can be seen in the lengthening of the word “ready” compared to the unaltered sentence. The shortening toward the end of the early sentence can be seen in the “-arlie go” portion of the sentence. The Altered (High-rate) sentence has smaller periods of duration change that are less salient visually, but the shortening and lengthening can be seen, for example, within the first word of the sentence, where the first syllable “Rea-” is shortened and the second syllable “-dy” is lengthened. This modulation process ensured that for every 50 ms frame that was lengthened, another frame was shortened by the same amount, thus keeping the overall duration of the rhythmically altered portion of the sentence identical to the unaltered version (see the red box in Figure 2) regardless of the rhythm alteration applied.

The phase of the modulator determined the proportion of compression or expansion at a given point in the cycle. The initial phase of the modulator was randomly drawn from eight different values (0 to 7π/4, with a π/4 spacing) with equal probabilities.

### Procedure

Listeners were seated in a sound attenuated booth in front of a computer monitor while auditory signals were presented diotically through insert headphones. During the temporal-shift detection task, participants listened to an intact sentence followed by two presentations of the same sentence in which two consecutive late-occurring words (“white six”) were replaced with a silent period that was either equal to, shorter, or longer than the duration of those words in the intact sentences. The task was to identify which of the two comparison sentences with gaps was the one with the temporally shifted final-word onset. The order of the three rhythm conditions (unaltered, low-rate, high-rate) was randomly drawn for each participant. Each rhythm condition consisted of three consecutive blocks of 40 trials. Each block included an equal number of early- and late-onset trials presented in a random order, preventing participants from identifying the altered sentence based on the total sentence duration. Total testing time for each subject was approximately 3 h in two sessions, with neither session exceeding 2 h. The gap deviations (Δ*T*/*T*) for the early- and late-onset trials within a block were varied using two interleaved 2-down 1-up adaptive tracks. To create an easily detected starting point, the initial Δ*T*/*T* values were 1.0 (i.e., a 100% decrease in the standard gap, resulting in no gap) for the early-onset condition and 1.5 (i.e., a 150% increase in the standard gap) for the late-onset conditions. The gap deviation was bounded between 0 and 1 for the early-onset condition, and between 0 and 2 for the late-onset condition. The step size was half the gap deviation until the second reversal and ¼ of the gap deviation for the remainder of the block. When the gap deviation reached the upper or lower bound, that value was repeated until two correct responses caused a change in the opposite direction. In each block, reversals were tracked separately for early- and late-onset trials. A temporal-shift detection threshold was estimated for each of the early- and late-onset trials for each block by taking the average gap deviation of the last four reversals, and the mean threshold across three trial blocks was computed for each subject in each condition.

## Results

**Figure 3:**
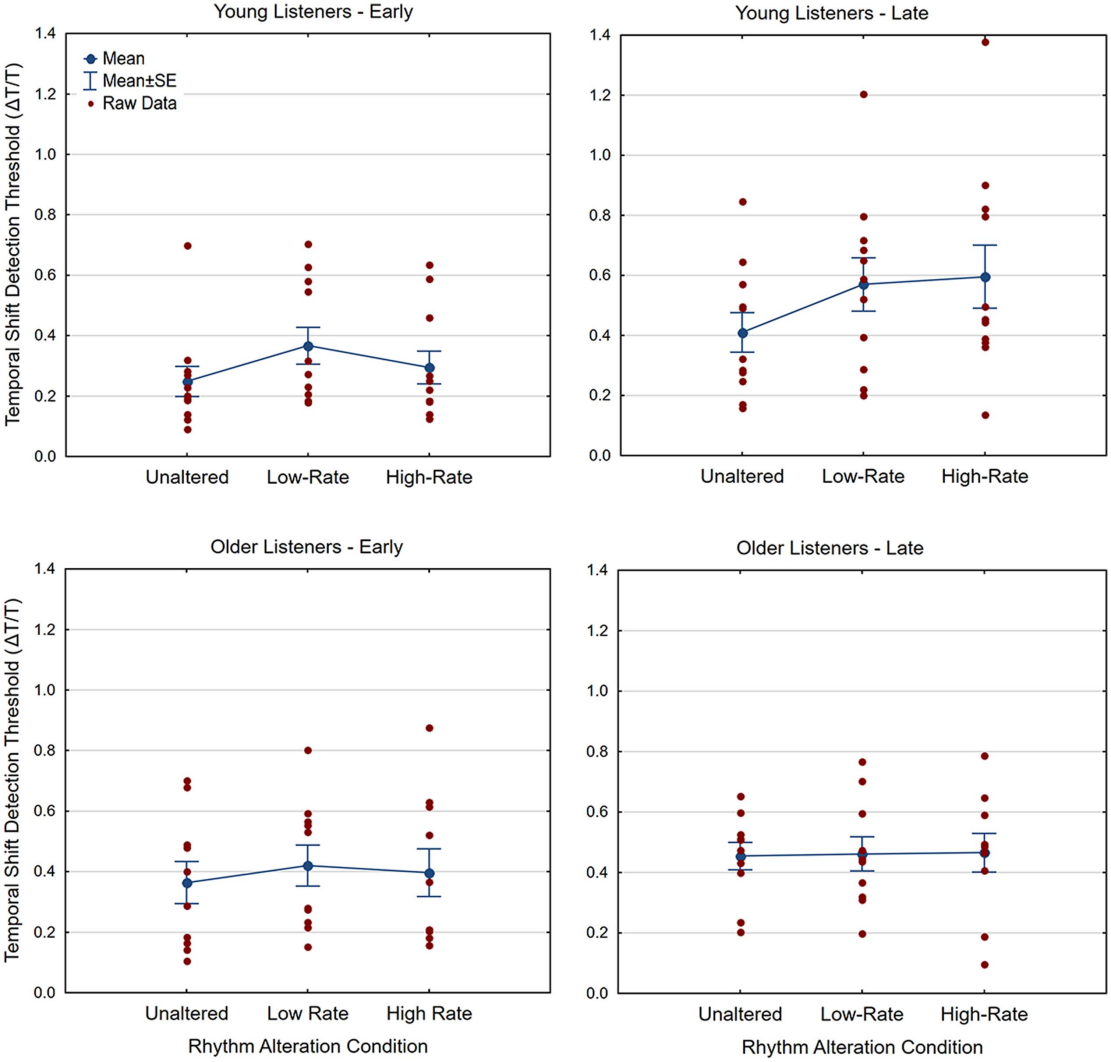
Temporal-shift detection thresholds (where a lower threshold indicates more accurate shift detection) for the young **(upper panels)** and older **(lower panels)** listener groups, plotted for all three rhythm conditions. Thresholds (ΔT/T) for the early-onset conditions are shown in the panels on the left, with late-onset thresholds on the right. Mean thresholds are shown by blue dots (connected by lines) and thresholds for all individual subjects are shown by red dots. Error bars represent the standard error of the mean.

Figure 3 shows temporal-shift detection thresholds (Δ*T*/*T*) for early and late temporal shifts for the three rhythm modulation conditions for the young and older listener groups. A 2 (Age: Young vs. Older) × 3 (Rhythm Modulation: Unaltered, Altered Low-Rate, Altered High-Rate) × 2 (Temporal-Shift Direction: Early onset vs. Late onset) mixed-measures analyses of variance (ANOVA) on detection thresholds revealed no main effect of Age, *F*(1, 19) = 0.024, *p* = 0.88, *η*^2^ = 0.001, but main effects of Rhythm Modulation, *F*(1.46, 27.78) = 6.14, *p* = 0.011, *η*^2^ = 0.241, and Temporal Shift Direction, *F*(1, 19) = 16.22, *p* = 0.001, *η*^2^ = 0.460, as well as an interaction between Temporal Shift Direction and Age [*F*(1, 19) = 4.616, *p* = 0.045, *η*^2^ = 0.191]. There were no other interactions (all *p*’s > 0.19).

The lack of a main effect of age shows that thresholds, overall, do not reliably differ between the young adult group (*M* = 0.42, *SD* = 0.20, 95% CI = 0.29–0.54) and the older adult group (*M* = 0.43, *SD* = 0.17, 95% CI = 0.30–0.54). With respect to the main effect of Rhythm Modulation, post-hoc t-tests show that detection thresholds are lower (better) for the unaltered rhythm modulation condition (*M* = 0.37, *SD* = 0.17) than those for the altered low-rate condition [*M* = 0.46, *SD* = 0.21, *t*(20) = −3.05, *p* = 0.006, Cohen’s *d* = −0.66] and those for the altered high-rate conditions [*M* = 0.44, *SD* = 0.22, *t*(20) = −3.67, *p* = 0.002, Cohen’s *d* = −0.80], but that thresholds for the two altered rhythm conditions do not significantly differ, [*t*(20) = 0.53, *p* = 0.6, Cohen’s *d* = 0.12]. With respect to the main effect of Temporal Shift Direction, detection thresholds are reliably lower (better) for early onsets (*M* = 0.35, *SD* = 0.19) than for late onsets (*M* = 0.50, *SD* = 0.22). Post-hoc paired *t*-tests investigating the interaction between Direction and Age reveal that young adults show significantly lower thresholds for early onsets compared to late onsets [Early, *M* = 0.30, *SD* = 0.17; Late, *M* = 0.53, *SD* = 0.27; *t*(10) = −4.85, *p* = 0.001, Cohen’s *d* = −1.46], but that older adults show no difference in temporal-shift detection thresholds between the early and late onset conditions [Early, *M* = 0.39, *SD* = 0.22; Late, *M* = 0.46, *SD* = 0.16; *t*(9) = −1.2, *p* = 0.26, Cohen’s *d* = −0.38].

### Individual differences among listeners

Although this is not an individual differences study, an examination of the performance of individual participants can help with the interpretation of the results. This is especially true in the current study, due to the relatively large individual differences within each age group. Although there was no main effect of age in this study, there was a significant interaction between Age and Temporal Shift Direction: only the younger group had significantly higher thresholds for late than for early onsets.

**Figure 4:**
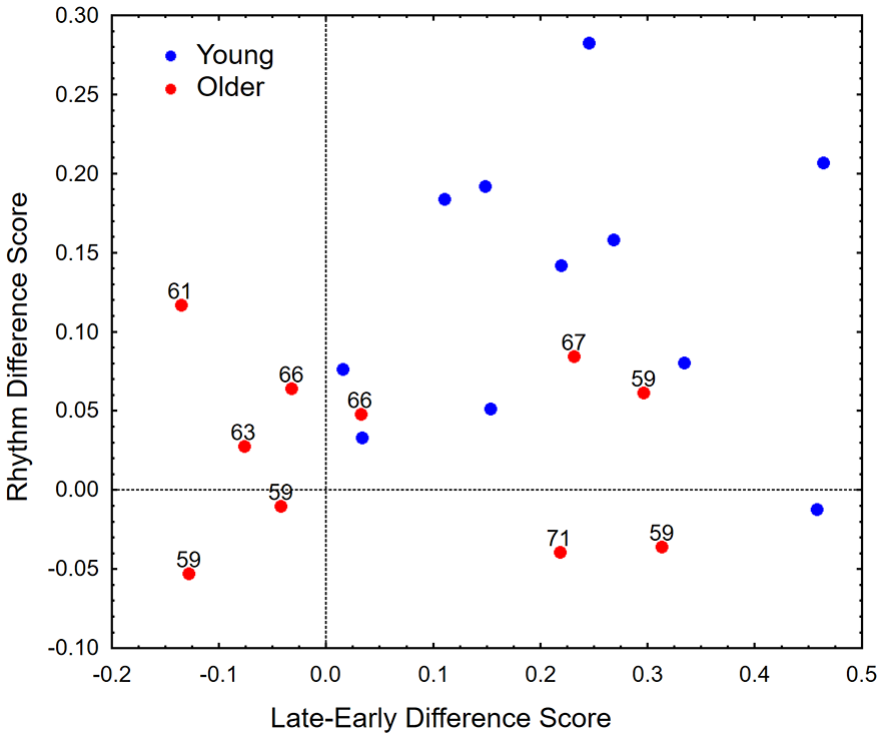
Late-early difference scores (mean of thresholds for late onsets minus the mean of thresholds for early onsets) and rhythm difference scores (mean of thresholds for the altered rhythm conditions minus the mean of thresholds for the unaltered rhythm condition) for each subject. Ages are shown for the older subjects.

The individual differences in performance in this study are shown in Figure 4, which presents the data in terms a late-early difference score (mean of late-onset thresholds minus the mean of early onset thresholds) on the abscissa and a rhythm difference score (mean altered-rhythm thresholds minus the mean unaltered-rhythm thresholds) on the ordinate. It can be seen that among the older listeners (red dots, with ages indicated), four performed more like the younger listeners (blue dots) in terms of the difference between early and late thresholds. However, the rhythm difference scores for these older subjects were lower than those for many of the younger subjects with similar late-early difference scores, indicating somewhat less sensitivity to the rhythm manipulation despite the larger late-early difference scores.

The four subjects in the older group with greater late-early difference scores (like those of the younger subjects) included the two oldest subjects (67 and 71 years), clearly showing that age alone does not account for their performance. To further examine individual differences among the older subjects we utilized data from an earlier study in which nine of the 10 older subjects had participated. The earlier study included a large test battery focusing on rhythm and speech perception. Three temporal tasks were selected from the battery, based on their focus on temporal and rhythm processing, and a working-memory measure was included to evaluate cognitive abilities. The first temporal task was a gap detection task (GAP), where listeners were asked to detect a gap (which varied over trials) in the middle of a 750-ms gaussian noise signal. The second was a rhythm discrimination (RD) task, where listeners made a same/different judgment about two similar rhythms formed by sequences of 6–8 tone pulses with temporal patterns defined by the sequence of tone-pulse intervals. The third was a synchronization and continuation (S&C) tapping task, where listeners tapped in synchrony with an isochronous auditory sequence, presented at different tempi, and then continued tapping at the same tempo after the stimulus stopped. Performance measures for these tasks were GAP: percent correct; RD: d-prime; S&C: the slope parameter of a linear regression that captures the central (non-motor) variability in tapped rhythm. The working-memory measure was from a working-memory test battery (Lewandowsky et al., 2010) consisting of three working memory tests: Memory updating, Sentence span, and Spatial short-term memory. The mean performance across all three working-memory (WM) tasks was used as a general measure of an important cognitive ability for the older participants. The tasks from the earlier study are described in more detail in Supplementary Appendix.

A correlation analysis including the GAP, S&C, RD, and WM scores from the earlier study and performance measures from the current study (late-early difference score, rhythm difference score, and overall mean performance) was conducted using the threshold data for the nine older subjects who had participated in the earlier study. The correlations and significance levels are shown in Table 1.

**Table 1:** Correlations between measures from the temporal-shift detection task and measures of temporal abilities and working memory from an earlier study, for the older participants in this study.

	Mean threshold	Late-early difference	Rhythm difference
Gap discrimination (GAP)	0.69*	0.19	−0.56
Tapping variability (S&C)	0.70*	0.20	−0.58
Rhythm discrimination (RD)	0.35	0.74*	0.19
Working memory (WM)	0.81**	0.46	−0.41

For ease of interpretation, the signs of all correlations have been set so that positive correlations indicate that better performance on a task from the earlier study (i.e., higher percent correct, higher d-prime, or lower variability) is associated with better performance (lower mean thresholds) or larger difference scores in the current study.

*p < 0.05, **p < 0.01.

**Figure 5:**
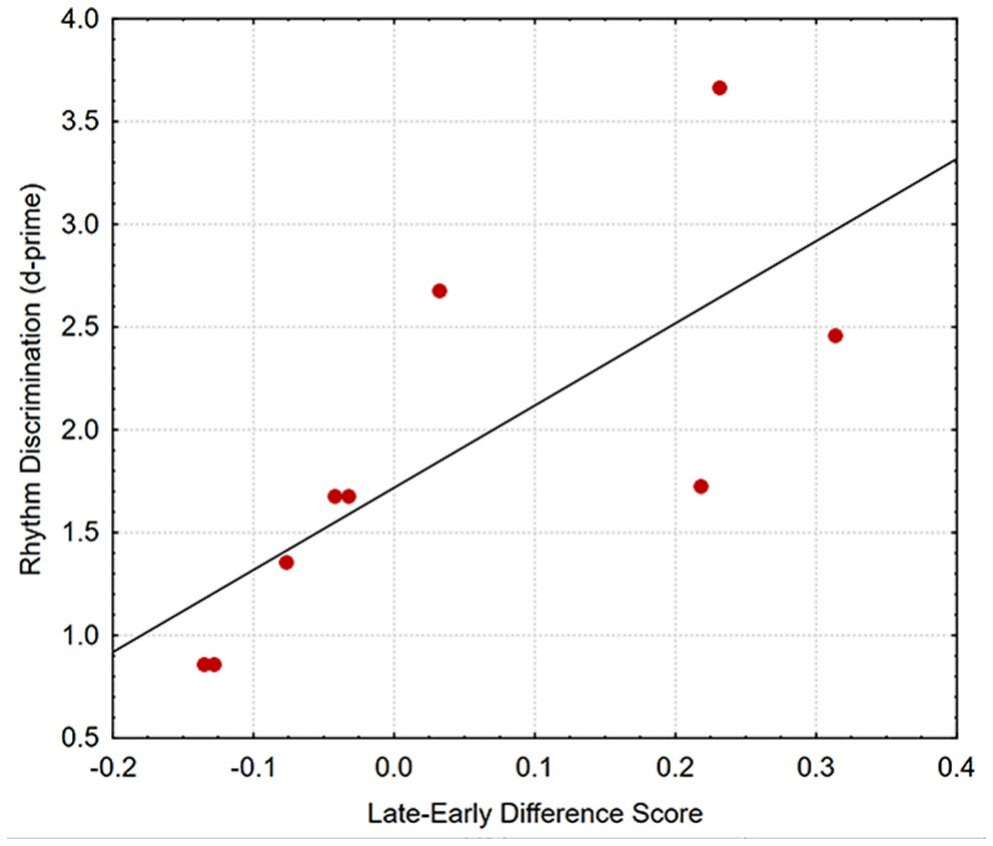
Scatterplot showing the correlation between performance in the rhythm discrimination task and the late-early difference scores (the difference between thresholds for late and early onsets) for the older listeners.

As seen in Table 1, three measures from the earlier study (GAP, S&C, and WM) were highly correlated with mean thresholds across all conditions in the present study. However, the one measure that was not correlated with overall performance (RD), was the only measure that was significantly correlated with the Late-Early Difference score. (The scatterplot in Figure 5 shows an orderly association between these two measures.) The difference in performance between altered and unaltered rhythm was not significantly correlated with any of the measures from the earlier study. These results show that the differential sensitivity to early vs. late onsets (which was the only age-related effect observed in this study) is related to rhythm-discrimination ability, but not to other factors (as measured by GAP, S&C, and WM tasks) that are related to overall performance.

## Discussion

### Temporal-shift detection among young and older listeners

The main purpose of this study was to examine young and older listeners’ reliance on speech rhythm in predicting the onset of upcoming speech events. Participants detected temporal deviations in the onset of the final word in spoken sentences with and without alterations of the natural speech rhythm in the early portion of the sentences. Both young and older listeners were better at detecting temporal deviations (early and late onsets of the final word) when the rhythm of the early portion of the sentences was intact than when it was altered, and there was no difference in their overall performance levels. However, the younger group was significantly less accurate with late onsets than with early onsets, while older listeners were unaffected by the direction of the temporal shift.

These findings are consistent with a DAT framework and an entrainment timing model in which listeners’ internal (attentional) rhythm is entrained by an external, rhythmic stimulus (Jones, 1976; Jones and Boltz, 1989; McAuley and Kidd, 1998; Large and Jones, 1999; McAuley and Jones, 2003). The alteration of the natural rhythm disrupts listener entrainment and weakens the temporal expectations needed for optimal anticipation of the onset of upcoming speech events. In this experiment, listeners’ temporal expectations for the onset of the final word (“now”) are less precise without an intact natural rhythmic structure, making it more difficult to detect the temporal shift in the altered test sentences. This result is in line with existing literature showing that listeners have more difficulty with the perception of auditory patterns, both speech and nonspeech, with altered or irregular rhythmic structures (Rimmele et al., 2012; Aubanel et al., 2016; Wang et al., 2018; Shen and Pearson, 2019; McAuley et al., 2020, 2021). The present demonstration of the effect of rhythmic context on timing judgments in speech reinforces the notion that irregular or altered rhythms have a negative impact on understanding because of their effect on temporal expectancies. That irregular rhythms affect both temporal predictions (in the current study) and speech understanding (in previous work) suggests that entrainment to speech rhythm underlies both phenomena: a consistent, natural speech rhythm promotes entrainment which guides temporal expectancies which, in turn, facilitate the perception of speech events that occur at expected times.

Despite no overall differences in thresholds between the two age groups, there were significant differences in sensitivity to temporal deviations between young and older listeners. Young listeners demonstrated a significant asymmetry in detection thresholds, with consistently worse performance for late onsets than for early onsets. Related asymmetries have been demonstrated in previous work examining responses to deviations from an established temporal pattern in an acoustic signal. This is true for both the detection of a temporal deviation and for the perception of a stimulus presented earlier or later than expected. For example, in a study of the effect of deviations from expected timing on tempo judgments with isochronous tone sequences, McAuley and Kidd (1998) found that with relatively fast tempos, subjects were better at detecting tempo increases when a sequence was presented earlier than expected, but they were better at detecting tempo decreases with late-onset sequences. McAuley and Fromboluti (2014) found that the durations of unexpectedly early events were underestimated, while the durations of late events were overestimated. Another type of early/late asymmetry has been found in studies of the detection of temporally deviant onsets in isochronous sequences of clicks or brief tones: Listeners are differentially sensitive to early and late onsets, and relative performance for early and late onsets varies with the tempo of the sequence, with a slight advantage for early events at slower tempos (see Friberg and Sundberg, 1995). In a study of temporal-order judgment with auditory–visual pairs, Di Luca and Rhodes (2016) found that earlier-than-expected events were perceptually delayed, while late events were perceptually accelerated. An early/late asymmetry has also been observed in tapping tasks, where an unexpectedly early event in a guiding sequence disrupts tapping more than a late event, particularly at short inter-onset intervals (Repp, 2011; Repp and Moseley, 2012).

In the current study, the early-late asymmetry is consistent with expectations based on speech rhythm: a slowing tempo is often expected at the end of a sentence, whereas a sudden tempo increase at the end of a sentence is less likely (Delattre, 1966; Oller, 1973; Cooper and Paccia-Cooper, 1980), thus making the timing of a late onset seem correct and an early onset surprising and more salient. This type of asymmetry is consistent with a dynamic-attending account in which expectations are linked to an attentional rhythm that is guided by external timing, but also influenced by other factors (such as temporal expectations in a given context (e.g., speech), or pattern tempo relative to a preferred tempo). In addition to their speech-rate expectations, young listeners may be less sensitive to late events because of an asymmetry in the attentional pulse that facilitates perception of events that occur at expected temporal locations (see Jones, 1976; Large and Jones, 1999). That is, early events (earlier than expected) never fall within an attentional pulse, but attention may be sustained beyond the expected event onset, especially when pattern slowing is expected (as with phrase-final slowing in speech and music). Early events that do not coincide with an attentional pulse are generally less well resolved, but the “surprise” of an unexpectedly early event can attract attention and enhance the detection of early events (see Large and Jones, 1999). However, despite the enhanced detection, the lack of attentional focus generally results in poorer resolving power and poorer identification of early events. Late-occurring events that are only slightly late can fall within the attentional pulse, especially in a context in which slowing may be expected. These events lack the surprise-based salience of early events, and the timing delay may not be as noticeable, but they are often well-resolved because listeners are still attentionally prepared.

The lack of an early/late event asymmetry in the older group suggests that there may be a decline in sensitivity to speech rhythms with age. That older listeners were affected by rhythmic alteration indicates that they were still attuned to speech rhythms and could make use of them, but the lack of an early-onset advantage suggests a weakening of speech-based expectations that result in an asymmetry in the sensitivity to early and late sentence-final events.

Although as a group, the older listeners did not show a significant difference in their detection of early and late events, some older listeners did show an early/late asymmetry like that seen with young listeners. And those older listeners who did show the asymmetry also showed better rhythm-discrimination abilities with tone sequences in an earlier study (described above and in Supplementary Appendix). This suggests that not all older listeners experience the same decrease in sensitivity to speech rhythms. Although the basis for this performance difference within the older group in this study cannot be determined by the current findings, the results provide some encouragement for the continuing search for factors (e.g., auditory training, musical experience, exercise) that may reduce a decline in listening abilities with age. If, as the data suggest, age-related declines in listening abilities are related to a decrease in the ability to attentionally synchronize with speech rhythms (or external rhythms in general), then more experience with active listening to rhythmic stimuli (such as speech and music) should help preserve listening abilities as people age. Further research with a wider range of speech materials and larger groups of listeners with different listening experience will provide a better understanding of how sensitivity to speech rhythms affects speech perception and how that sensitivity changes with age.

## Conclusion

Both young and older listeners were shown to rely on the rhythm of a spoken sentence when judging whether a sentence-final word was presented at the correct time after a brief muting of the sentence (a silent period replacing two words prior to the final word). This extends findings from earlier work showing that altering the natural rhythm of spoken sentences adversely affects speech understanding. The same type of rhythm alterations that led to poorer speech understanding in the earlier studies resulted in poorer detection of changes in the onset of the final word in the present study. This suggests that a decreased ability to predict the onset of upcoming speech events, resulting from an alteration of the natural speech rhythm, at least partly accounts for the poorer speech recognition performance observed with altered speech rhythms. These findings are consistent with dynamic attending theory, which proposes that speech understanding depends on the entrainment of attentional rhythms to speech rhythms, resulting in a facilitation of the perception of speech events that occur at expected times.

The findings also showed that young listeners’ temporal expectancies differed from those in the older group. Despite similar overall thresholds, young listeners were significantly worse at detecting late onsets of the final word in the sentence than early onsets, while the older group showed no significant difference in thresholds for early and late onsets. This suggests a greater reliance on speech-based expectancies with the younger listeners, whose performance was more consistent with an expectation of a slowing tempo at the end of a sentence. However, that some older listeners (those who performed better on a rhythm-discrimination task in an earlier study) showed an early-late asymmetry, like that observed with the younger group, suggests that not all older listeners undergo the same age-related change in their ability to use speech rhythms to guide temporal expectancies and facilitate speech understanding.

## Data availability statement

The raw data supporting the conclusions of this article will be made available by the authors, without undue reservation.

## Ethics statement

The studies involving human participants were reviewed and approved by the Institutional Review Board, Indiana University. The patients/participants provided their written informed consent to participate in this study.

## Author contributions

GK and JM conceived the study. DP wrote the first draft and performed the statistical analysis with YS. All authors contributed to the design and to the manuscript revision, and all approved the submitted version.

## Funding

This research was supported by the NIH (Grant Nos. R01DC013538 to PIs: GK and JM and R01DC017988 to PI: YS).

## Conflict of interest

The authors declare that the research was conducted in the absence of any commercial or financial relationships that could be construed as a potential conflict of interest.

## Publisher’s note

All claims expressed in this article are solely those of the authors and do not necessarily represent those of their affiliated organizations, or those of the publisher, the editors and the reviewers. Any product that may be evaluated in this article, or claim that may be made by its manufacturer, is not guaranteed or endorsed by the publisher.

## References

[ref1] Ahissar E. Nagarajan S. Ahissar M. Protopapas A. Mahncke H. Merzenich M. M. (2001). Speech comprehension is correlated with temporal response patterns recorded from auditory cortex. Proc. Natl. Acad. Sci. U. S. A. 98, 13367–13372. doi: 10.1073/pnas.201400998, PMID: 11698688 PMC60877

[ref2] Akeroyd M. A. (2008). Are individual differences in speech reception related to individual differences in cognitive ability? A survey of twenty experimental studies with normal and hearing-impaired adults. Int. J. Audiol. 47, S53–S71. doi: 10.1080/14992020802301142, PMID: 19012113

[ref3] ANSI (2004). S3.6–2004, specification for audiometers. New York, NY: ANSI.

[ref4] Aubanel V. Davis C. Kim J. (2016). Exploring the role of brain oscillations in speech perception in noise: intelligibility of isochronously retimed speech. Front. Syst. Neurosci. 10:430. doi: 10.3389/fnhum.2016.00430 PMC5006149 27630552

[ref5] Baese-Berk M. M. Dilley L. C. Henry M. J. Vinke L. Banzina E. (2019). Not just a function of function words: distal speech rate influences perception of prosodically weak syllables. Atten. Percept. Psychol. 81, 571–589. doi: 10.3758/s13414-018-1626-4, PMID: 30488190

[ref6] Bolia R. S. Nelson W. T. Ericson M. A. Simpson B. D. (2000). A speech corpus for multitalker communications research. J. Acoust. Soc. Am. 107, 1065–1066. doi: 10.1121/1.428288, PMID: 10687719

[ref7] Carroll R. Warzybok A. Kollmeier B. Ruigendijk E. (2016). Age-related differences in lexical access relate to speech recognition in noise. Front. Psychol. 7:990. doi: 10.3389/fpsyg.2016.00990 27458400 PMC4930932

[ref8] Cooper W. E. Paccia-Cooper J. (1980) Syntax and speech. Cambridge: Harvard University Press.

[ref9] Cutler A. Dahan D. van Donselaar W. (1997). Prosody in the comprehension of spoken language: a literature review. Lang. Speech 40, 141–201. doi: 10.1177/002383099704000203 9509577

[ref10] Cutler A. Mehler J. (1993). The periodicity bias. J. Phon. 21, 103–108. doi: 10.1016/S0095-4470(19)31323-3

[ref11] Cutler A. Swinney D. A. (1987). Prosody and the development of comprehension. J. Child Lang. 14, 145–167. doi: 10.1017/S0305000900012782 3558521

[ref12] Delattre P. (1966). A comparison of syllable length conditioning among languages. Int. Rev. Appl. Linguist. 4, 183–198.

[ref13] Di Luca M. Rhodes D. (2016). Optimal perceived timing: integrating sensory information with dynamically updated expectations. Sci. Rep. 6:28563. doi: 10.1038/srep28563, PMID: 27385184 PMC4935895

[ref14] Dilley L. C. McAuley J. D. (2008). Distal prosodic context affects word segmentation and lexical processing. J. Mem. Lang. 59, 294–311. doi: 10.1016/j.jml.2008.06.006

[ref15] Dubno J. R. Horwitz A. R. Ahlstrom J. B. (2002). Benefit of modulated maskers for speech recognition by younger and older adults with normal hearing. J. Acoust. Soc. Am. 111, 2897–2907. doi: 10.1121/1.1480421, PMID: 12083223

[ref16] Fitzgibbons P. J. Gordon-Salant S. (1994). Age effects on measures of auditory duration discrimination. J. Speech Lang. Hear. Res. 37, 662–670. doi: 10.1044/jshr.3703.662 8084196

[ref17] Fitzgibbons P. J. Gordon-Salant S. (1995). Age effects on duration discrimination with simple and complex stimuli. J. Acoust. Soc. Am. 98, 3140–3145. doi: 10.1121/1.413803, PMID: 8550939

[ref18] Fitzgibbons P. J. Gordon-Salant S. (2001). Aging and temporal discrimination in auditory sequences. J. Acoust. Soc. Am. 109, 2955–2963. doi: 10.1121/1.1371760, PMID: 11425137

[ref19] Fitzgibbons P. J. Gordon-Salant S. (2004). Age effects on discrimination of timing in auditory sequences. J. Acoust. Soc. Am. 116, 1126–1134. doi: 10.1121/1.1765192, PMID: 15376678

[ref20] Fitzgibbons P. J. Gordon-Salant S. (2010a). “Behavioral studies with aging humans: hearing sensitivity and psychoacoustics” in The aging auditory system. ed. Gordon-Salant S., vol. 34 (New York, NY: Springer-Verlag), 111–134.

[ref21] Fitzgibbons P. J. Gordon-Salant S. (2010b). Age-related differences in discrimination of temporal intervals in accented tone sequences. Hear. Res. 264, 41–47. doi: 10.1016/j.heares.2009.11.008, PMID: 19931608 PMC2868081

[ref22] Fitzgibbons P. J. Gordon-Salant S. (2011). Age effects in discrimination of repeating sequence intervals. J. Acoust. Soc. Am. 129, 1490–1500. doi: 10.1121/1.3533728, PMID: 21428513 PMC3078028

[ref23] Fletcher J. (2010). “The prosody of speech: timing and rhythm” in The handbook of phonetic sciences. 2nd ed. eds. Hardcastle W. J. Laver J. Gibbon F. E. (Wiley Online Library), 521–602.

[ref24] Folstein M. F. Folstein S. E. McHugh P. R. (1975). Mini-mental state: a practical method for grading the cognitive state of patients for the clinician. J. Psychiatr. Res. 12, 189–198. doi: 10.1016/0022-3956(75)90026-6 1202204

[ref25] Friberg A. Sundberg J. (1995). Time discrimination in a monotonic, isochronous sequence. J. Acoust. Soc. Am. 98, 2524–2531. doi: 10.1121/1.413218

[ref26] Frisina D. R. Frisina R. D. (1997). Speech recognition in noise and presbycusis: relations to possible neural mechanisms. Hear. Res. 106, 95–104. doi: 10.1016/S0378-5955(97)00006-3, PMID: 9112109

[ref27] Füllgrabe C. Moore B. C. Stone M. A. (2015). Age-group differences in speech identification despite matched audiometrically normal hearing: contributions from auditory temporal processing and cognition. Front. Aging Neurosci. 6:347. doi: 10.3389/fnagi.2014.00347 25628563 PMC4292733

[ref28] Gallego Hiroyasu E. M. Yotsumoto Y. (2020). Older adults preserve accuracy but not precision in explicit and implicit rhythmic timing. PLoS One 15:e0240863. doi: 10.1371/journal.pone.0240863, PMID: 33075063 PMC7571673

[ref29] George E. L. Festen J. M. Houtgast T. (2006). Factors affecting masking release for speech in modulated noise for normal-hearing and hearing-impaired listeners. J. Acoust. Soc. Am. 120, 2295–2311. doi: 10.1121/1.2266530, PMID: 17069325

[ref30] George E. L. Zekveld A. A. Kramer S. E. Goverts S. T. Festen J. M. Houtgast T. (2007). Auditory and nonauditory factors affecting speech reception in noise by older listeners. J. Acoust. Soc. Am. 121, 2362–2375. doi: 10.1121/1.2642072, PMID: 17471748

[ref31] Gieseler A. Tahden M. A. Thiel C. M. Wagener K. C. Meis M. Colonius H. (2017). Auditory and non-auditory contributions for unaided speech recognition in noise as a function of hearing aid use. Front. Psychol. 8:219. doi: 10.3389/fpsyg.2017.00219 28270784 PMC5318449

[ref32] Giraud A. L. Poeppel D. (2012). Cortical oscillations and speech processing: emerging computational principles and operations. Nat. Neurosci. 15, 511–517. doi: 10.1038/nn.3063, PMID: 22426255 PMC4461038

[ref33] Golumbic E. M. Z. Poeppel D. Schroeder C. E. (2012). Temporal context in speech processing and attentional stream selection: a behavioral and neural perspective. Brain Lang. 122, 151–161. doi: 10.1016/j.bandl.2011.12.010, PMID: 22285024 PMC3340429

[ref34] Gordon-Salant S. (2005). Hearing loss and aging: new research findings and clinical implications. J. Rehabil. Res. Dev. 42, 9–24. doi: 10.1682/JRRD.2005.01.0006, PMID: 16470462

[ref35] Gordon-Salant S. Fitzgibbons P. J. (1993). Temporal factors and speech recognition performance in young and elderly listeners. J. Speech Lang. Hear. Res. 36, 1276–1285. doi: 10.1044/jshr.3606.1276 8114494

[ref36] Gordon-Salant S. Fitzgibbons P. J. (1999). Profile of auditory temporal processing in older listeners. J. Speech Lang. Hear. Res. 42, 300–311. doi: 10.1044/jslhr.4202.300, PMID: 10229448

[ref37] Gordon-Salant S. Fitzgibbons P. J. (2004). Effects of stimulus and noise rate variability on speech perception by younger and older adults. J. Acoust. Soc. Am. 115, 1808–1817. doi: 10.1121/1.1645249, PMID: 15101658

[ref38] Halpern A. R. Darwin C. J. (1982). Duration discrimination in a series of rhythmic events. Percept. Psychophys. 31, 86–89. doi: 10.3758/BF03206204, PMID: 7070941

[ref39] Holmes E. Griffiths T. D. (2019). ‘Normal’ hearing thresholds and fundamental auditory grouping processes predict difficulties with speech-in-noise perception. Sci. Rep. 9, 1–11. doi: 10.1038/s41598-019-53353-5 31728002 PMC6856372

[ref40] Houtgast T. Festen J. M. (2008). On the auditory and cognitive functions that may explain an individual’s elevation of the speech reception threshold in noise. Int. J. Audiol. 47, 287–295. doi: 10.1080/14992020802127109, PMID: 18569101

[ref41] Hoyte K. J. Brownell H. Wingfield A. (2009). Components of speech prosody and their use in detection of syntactic structure by older adults. Exp. Aging Res. 35, 129–151. doi: 10.1080/03610730802565091, PMID: 19173106 PMC2867101

[ref42] Humes L. E. (2007). The contributions of audibility and cognitive factors to the benefit provided by amplified speech to older adults. J. Am. Acad. Audiol. 18, 590–603. doi: 10.3766/jaaa.18.7.6, PMID: 18236646

[ref43] Humes L. E. (2021). Longitudinal changes in auditory and cognitive function in middle-aged and older adults. J. Speech Lang. Hear. Res. 64, 230–249. doi: 10.1044/2020_JSLHR-20-00274, PMID: 33400551 PMC8608226

[ref44] Humes L. E. Busey T. A. Craig J. C. Kewley-Port D. (2009). The effects of age on sensory thresholds and temporal gap detection in hearing, vision, and touch. Atten. Percept. Psychol. 71, 860–871. doi: 10.3758/APP.71.4.860, PMID: 19429964 PMC2826883

[ref45] Humes L. E. Dubno J. R. (2010). “Factors affecting speech understanding in older adults” in The aging auditory system: perceptual characterization and neural bases of Presbycusis. eds. Gordon-Salant S. Frisina R. D. Popper A. N. Fay R. R. (New York, NY: Springer), 211–257.

[ref46] Humes L. E. Kidd G. R. Lentz J. J. (2013). Auditory and cognitive factors underlying individual differences in aided speech-understanding among older adults. Front. Syst. Neurosci. 7:55. doi: 10.3389/fnsys.2013.00055 24098273 PMC3787592

[ref47] Humes L. E. Watson B. U. Christensen L. A. Cokely C. G. Halling D. C. Lee L. (1994). Factors associated with individual differences in clinical measures of speech recognition among the elderly. J. Speech Lang. Hear. Res. 37, 465–474. doi: 10.1044/jshr.3702.465, PMID: 8028328

[ref48] Jones M. R. (1976). Time, our lost dimension: toward a new theory of perception, attention, and memory. Psychol. Rev. 83, 323–355. doi: 10.1037/0033-295X.83.5.323, PMID: 794904

[ref49] Jones M. R. Boltz M. (1989). Dynamic attending and responses to time. Psychol. Rev. 96, 459–491. doi: 10.1037/0033-295X.96.3.459 2756068

[ref50] Jones M. R. Mcauley J. D. (2005). Time judgments in global temporal contexts. Percept. Psychophys. 67, 398–417. doi: 10.3758/BF03193320, PMID: 16119390

[ref51] Jones M. R. Moynihan H. MacKenzie N. Puente J. (2002). Temporal aspects of stimulus-driven attending in dynamic arrays. Psychol. Sci. 13, 313–319. doi: 10.1111/1467-9280.00458, PMID: 12137133

[ref52] Karlin J. E. (1942). A factorial study of auditory function. Psychometrika 7, 251–279. doi: 10.1007/BF02288628

[ref53] Kidd G. R. (1989). Articulatory-rate context effects in phoneme identification. J. Exp. Psychol. Hum. Percept. Perform. 15, 736–748. doi: 10.1037/0096-1523.15.4.736, PMID: 2531208

[ref54] Kidd G. R. Watson C. S. Gygi B. (2007). Individual differences in auditory abilities. J. Acoust. Soc. Am. 122, 418–435. doi: 10.1121/1.2743154 17614500

[ref55] Large E. W. Jones M. R. (1999). The dynamics of attending: how people track time-varying events. Psychol. Rev. 106, 119–159. doi: 10.1037/0033-295X.106.1.119

[ref56] Lewandowsky S. Oberauer K. Yang L. X. Ecker U. K. H. (2010). A working memory test battery for MATLAB. Behav. Res. Methods 42, 571–585. doi: 10.3758/brm.42.2.571, PMID: 20479189

[ref57] Lister J. Tarver K. (2004). Effect of age on silent gap discrimination in synthetic speech stimuli. J. Speech Lang. Hear. Res. 47, 257–268. doi: 10.1044/1092-4388(2004/021), PMID: 15157128

[ref58] Luo H. Poeppel D. (2007). Phase patterns of neuronal responses reliably discriminate speech in human auditory cortex. Neuron 54, 1001–1010. doi: 10.1016/j.neuron.2007.06.004, PMID: 17582338 PMC2703451

[ref59] Martin J. G. (1972). Rhythmic (hierarchical) versus serial structure in speech and other behavior. Psychol. Rev. 79, 487–509. doi: 10.1037/h0033467, PMID: 4634593

[ref60] McAuley J. D. Fromboluti E. K. (2014). Attentional entrainment and perceived event duration. Philos. Trans. R. Soc. B: Biol. Sci. 369:20130401. doi: 10.1098/rstb.2013.0401, PMID: 25385779 PMC4240968

[ref61] McAuley J. D. Jones M. R. (2003). Modeling effects of rhythmic context on perceived duration: a comparison of interval and entrainment approaches to short-interval timing. J. Exp. Psychol. Hum. Percept. Perform. 29, 1102–1125. doi: 10.1037/0096-1523.29.6.1102, PMID: 14640833

[ref62] McAuley J. D. Kidd G. R. (1998). Effect of deviations from temporal expectations on tempo discrimination of isochronous tone sequences. J. Exp. Psychol. Hum. Percept. Perform. 24, 1786–1800. doi: 10.1037/0096-1523.24.6.1786, PMID: 9861723

[ref63] McAuley J. D. Shen Y. Dec S. Kidd G. R. (2020). Altering the rhythm of target and background talkers differentially affects speech understanding. Atten. Percept. Psychol. 82, 3222–3233. doi: 10.3758/s13414-020-02064-5, PMID: 32458224 PMC10575213

[ref64] McAuley J. D. Shen Y. Smith T. Kidd G. R. (2021). Effects of speech-rhythm disruption on selective listening with a single background talker. Atten. Percept. Psychol. 83, 2229–2240. doi: 10.3758/s13414-021-02298-x PMC10612531 33782913

[ref65] Miller J. E. Carlson L. A. McAuley J. D. (2013). When what you hear influences when you see: listening to an auditory rhythm influences the temporal allocation of visual attention. Psychol. Sci. 24, 11–18. doi: 10.1177/0956797612446707, PMID: 23160202

[ref66] Morrill T. H. Dilley L. C. McAuley J. D. Pitt M. A. (2014). Distal rhythm influences whether or not listeners hear a word in continuous speech: support for a perceptual grouping hypothesis. Cognition 131, 69–74. doi: 10.1016/j.cognition.2013.12.006, PMID: 24457086

[ref67] Nuesse T. Steenken R. Neher T. Holube I. (2018). Exploring the link between cognitive abilities and speech recognition in the elderly under different listening conditions. Front. Psychol. 9:678. doi: 10.3389/fpsyg.2018.00678, PMID: 29867654 PMC5968383

[ref68] Oller D. K. (1973). The effect of position in utterance on speech segment duration in English. J. Acoust. Soc. Am. 54, 1235–1247. doi: 10.1121/1.1914393, PMID: 4765808

[ref69] Peelle J. E. Davis M. H. (2012). Neural oscillations carry speech rhythm through to comprehension. Front. Psychol. 3:320. doi: 10.3389/fpsyg.2012.00320 22973251 PMC3434440

[ref70] Pichora-Fuller M. K. Schneider B. A. Daneman M. (1995). How young and old adults listen to and remember speech in noise. J. Acoust. Soc. Am. 97, 593–608. doi: 10.1121/1.412282, PMID: 7860836

[ref71] Repp B. H. (2011). Tapping in synchrony with a perturbed metronome: the phase correction response to small and large phase shifts as a function of tempo. J. Mot. Behav. 43, 213–227. doi: 10.1080/00222895.2011.561377, PMID: 21480027

[ref72] Repp B. H. Moseley G. P. (2012). Anticipatory phase correction in sensorimotor synchronization. Hum. Mov. Sci. 31, 1118–1136. doi: 10.1016/j.humov.2011.11.001, PMID: 22230715

[ref73] Rimmele J. Schroger E. Bendixen A. (2012). Age-related changes in the use of regular patterns for auditory scene analysis. Hear. Res. 289, 98–107. doi: 10.1016/j.heares.2012.04.006, PMID: 22543088

[ref74] Schneider B. A. Daneman M. Murphy D. R. (2005). Speech comprehension difficulties in older adults: cognitive slowing or age-related changes in hearing? Psychol. Aging 20, 261–271. doi: 10.1037/0882-7974.20.2.261 16029090

[ref75] Schneider B. A. Pichora-Fuller M. K. Kowalchuk D. Lamb M. (1994). Gap detection and the precedence effect in young and old adults. J. Acoust. Soc. Am. 95, 980–991. doi: 10.1121/1.408403, PMID: 8132912

[ref76] Shen Y. Pearson D. V. (2019). Efficiency in glimpsing vowel sequences in fluctuating makers: effects of temporal fine structure and temporal regularity. J. Acoust. Soc. Am. 145, 2518–2529. doi: 10.1121/1.5098949, PMID: 31046353 PMC6491349

[ref77] Snell K. B. Frisina D. R. (2000). Relationships among age-related differences in gap detection and word recognition. J. Acoust. Soc. Am. 107, 1615–1626. doi: 10.1121/1.428446, PMID: 10738815

[ref78] Sommers M. S. Humes L. E. (1993). Auditory filter shapes in normal-hearing, noise-masked normal, and elderly listenersa. J. Acoust. Soc. Am. 93, 2903–2914. doi: 10.1121/1.405810, PMID: 8315154

[ref79] Surprenant A. M. Watson C. S. (2001). Individual differences in the processing of speech and nonspeech sounds by normal-hearing listeners. J. Acoust. Soc. Am. 110, 2085–2095. doi: 10.1121/1.1404973, PMID: 11681386

[ref80] Tierney A. Rosen S. Dick F. (2020). Speech-in-speech perception, nonverbal selective attention, and musical training. J. Exp. Psychol. Learn. Mem. Cogn. 46, 968–979. doi: 10.1037/xlm0000767, PMID: 31580123

[ref81] Vermiglio A. J. Soli S. D. Freed D. J. Fisher L. M. (2012). The relationship between high-frequency pure-tone hearing loss, hearing in noise test (HINT) thresholds, and the articulation index. J. Am. Acad. Audiol. 23, 779–788. doi: 10.3766/jaaa.23.10.4, PMID: 23169195

[ref82] Wang M. Kong L. Zhang C. Wu X. Li L. (2018). Speaking rhythmically improves speech recognition under “cocktail-party” conditions. J. Acoust. Soc. Am. 143, EL255–EL259. doi: 10.1121/1.5030518, PMID: 29716270

[ref83] Watson C. S. Kidd G. R. (2002). On the lack of association between basic auditory abilities, speech processing, and other cognitive skills. Semin. Hear. 23, 83–94. doi: 10.1055/s-2002-24978

[ref84] WHO (1991). Report of the informal working group on prevention of deafness and hearing impairment programme planning, Geneva, 18–21 June 1991. Geneva: World Health Organization.

