# Supplementary material for: Differential sensitivity to speech rhythms in young and older adults
Source: Front Psychol. 2023 May 12;14:1160236. doi: 10.3389/fpsyg.2023.1160236 (PMC10213510; doi:10.3389/fpsyg.2023.1160236)
Supplement: Supplementary file 1 [file Data_Sheet_1.pdf]

Differential sensitivity to speech rhythms in young and older adults

Dylan V. Pearson[1], Yi Shen[2], J. Devin McAuley[3] and Gary R. Kidd[1]

[1]Department of Speech, Language, and Hearing Sciences, Indiana University, Bloomington, IN, United States, [2]Department of Speech and Hearing Sciences, University of Washington, Seattle, WA, United States, [3]Department of Psychology, Michigan State University, East Lansing, MI, United States

Appendix: Metrics of temporal and rhythm processing

1. *Gap Detection*

The Test of Basic Auditory Capabilities (TBAC) is a battery of tests that measure a participants' auditory processing using a variety of discrimination tasks. Gap detection (GAP) is a subtask in the TBAC test battery (Watson et al, 1982; Christopherson and Humes, 1992; Kidd *et al*., 2007) where for 72 trials listeners are first presented the standard signal which is a 750-ms uninterrupted gaussian noise signal followed by two test signals. One of the test signals, determined randomly on each trial, is identical to the standard, while the other one is the standard signal interrupted by a silent gap. Listeners are tasked with identifying the sound contains the temporal gap. The gap duration ranges from 0.5 ms to 64 ms, and it is systematically varied such that the task becomes increasingly more difficult trial to trial. The result is reported as the overall percentage correct across all trials. This design allows for the percent correct score to reflect the length of duration listeners require to identify the presence of a gap in a noisy signal.

2. *Synchronization and Continuation Tapping Task*

In the synchronize and continue tapping tasks (S&C task), listeners were presented with an isochronous series of clicks and asked to tap a finger along with the click sequence in a synchronized fashion. After 42 presentations the click sequence ends while listeners were tasked with maintaining the same tapping rhythm as best they can for a duration of time equal to the duration of the isochronous click sequence. The variability in tapping rate in S&C may come from either variability in central timing processing or variability in the motor implementation of the movement. Our interest is in central timing processing so to

analyze the S&C results we utilized a component analysis method (Ivry and Corcos, 1993; Ivry and Hazeltine, 1995; McAuley *et al*., 2006) that uses the regression of tapping variability and target rate. This produces a slope value that corresponds to central timing variability in the slope analysis method (which was used in our analysis) and an intercept value corresponding to motor variability.

3. *Rhythm Discrimination Task*

For the rhythm discrimination task, listeners heard two consecutive identical standard rhythms followed by a comparison rhythm that was either identical to the standard or altered by swapping adjacent intervals somewhere in the rhythm (Povel and Essens, 1985; Grahn and Brett, 2007). Listeners were tasked to make a same/different discrimination of the comparison rhythm in both a simple rhythm condition and a complex rhythm condition (for our purposes we only used the simple rhythm data. The rhythmic sequences are constructed of five to seven intervals. These are selected from four interval lengths related to each other such that the length of each interval was equal to a multiple of the shortest interval. The length of the shortest interval was randomly generated between 220 and 270 ms.

Measures of Working Memory

(See Lewandowsky et al., (2010) for more details on these measures.)

Memory updating. At the start of each trial, subjects were presented with a sequence of from 3 to 5 digits. Each digit was surrounded by a square to mark its position on the screen. After all of the digits were presented, the squares remained on the screen and a different sequence of arithmetic operations (addition or subtraction, ranging from +7 to 7) appeared in each of the squares, one at a time. The subject's task was to remember the dig- its that appeared in each square and then perform the sequence of arithmetic operations presented in each of the squares. The subject was asked to indicate (using the keyboard) the final resulting value in each square after a sequence of from two to six sequential arithmetic operations. The test consisted of 15 trials with a randomly-generated sequence of set size (3–5 co-occurring series of operations) and number of operations (2–6) on each trial.

Because this test was challenging for older adults, some adjustments were made to the procedures to ensure that the task was well-understood, and to make it a bit less challenging. The number of practice trials was increased from two (the default) to four and the time between items (to be added or subtracted) was increased from 250 to 500 ms. The first two practice trials used a 3-s inter-item time to allow the experimenter to explain the required operations during the trial. Also, the default instructions were supplemented with a verbal explanation of the task that included a subject-paced simulated trial using cue cards to present the stimuli.

Sentence span. The "easy" version of the sentence-span task was used for this study. In this task, subjects were presented with an alternating sequence of simple sentences (3–6 words in length) and single letters on the computer screen. Subjects judged whether the sentence was true or false on each presentation, with 4 s allowed for responding. The letters required no response. After from four to eight sentence/letter presentations, subjects were asked to recall the letters in the order they were presented. The test consisted of 15 trials (after three practice trials) with three instances of each number of sentence/letter presentations.

Spatial short-term memory. This test assessed a subject's ability to recall the location of dots (filled circles) in a 10 × 10 grid. On each trial, an empty grid was presented and then a sequence of dots appeared in the grid. Each dot remained on the screen for ~1 s before it was removed, and the next dot appeared. From two to six dots were presented on each trial. After all of the dots had been presented (and removed), the subject was asked to indicate the relative position of the dots by touching (or pointing and clicking with a computer mouse) the cells within the grid. This test consisted of 30 trials (6 at each set size).

References

Christopherson, L. A., and Humes, L. E. (1992). Some psychometric properties of the Test of Basic Auditory Capabilities (TBAC). J. Speech Lang. Hear. Res., 35(4), 929-935.

Grahn, J. A., and Brett, M. (2007). Rhythm and beat perception in motor areas of the brain. J. Cogn. Neurosci., 19(5), 893-906.

Ivry, R., and Corcos, D. M. (1993). Slicing the variability pie: Component analysis of coordination and motor dysfunction. In K. M. Newell & D. M. Corcos (Eds.), Variability and motor control (pp. 415–447). Champaign, IL: Human Kinetics.

Ivry, R. B., and Hazeltine, R. E. (1995). Perception and production of temporal intervals across a range of durations: evidence for a common timing mechanism. J. Exp. Psychol. Hum. Percept. Perform., 21(1), 3.

Kidd, G. R., Watson, C. S., and Gygi, B. (2007). Individual differences in auditory abilities. J. Acoust. Soc. Am., 122(1), 418-435.

Lewandowsky, S., Oberauer, K., Yang, L. X., & Ecker, U. K. H. (2010). A working memory test battery for MATLAB. Behav. Res. Methods, 42(2), 571-585. doi:10.3758/brm.42.2.571.

McAuley, J. D., Jones, M. R., Holub, S., Johnston, H. M., and Miller, N. S. (2006). The time of our lives: life span development of timing and event tracking. J. Exp. Psychol. Gen., 135(3), 348.

Povel, D. J., and Essens, P. (1985). Perception of temporal patterns. Music Percept., 2(4), 411-440.

Watson, C. S., Johnson, D. M., Lehman, J. R., Kelly, W. J., and Jensen, J. K. (1982). An auditory discrimination test battery. J. Acoust. Soc. Am., 71(S1), S73-S73.
